# Multi-approach methods to predict cryptic carbapenem resistance mechanisms in *Klebsiella pneumoniae* detected in Central Italy

**DOI:** 10.3389/fmicb.2023.1242693

**Published:** 2023-08-28

**Authors:** Alessandra Cornacchia, Anna Janowicz, Gabriella Centorotola, Maria Antonietta Saletti, Sofia Chiatamone Ranieri, Massimo Ancora, Paola Ripà, Cesare Cammà, Francesco Pomilio, Alexandra Chiaverini

**Affiliations:** ^1^Istituto Zooprofilattico Sperimentale dell’Abruzzo e del Molise “G. Caporale”, Via Campo Boario, Teramo, Italy; ^2^Operative Unit of Clinical Pathology and Microbiology, Department of Services, ASL of Teramo, Teramo, Italy

**Keywords:** *Klebsiella pneumoniae*, carbapenems, bla_KPC_, whole genome sequencing, genome-wide association study

## Abstract

The rapid emergence of carbapenem-resistant *Klebsiella pneumoniae* (*Kp*) strains in diverse environmental niches, even outside of the clinical setting, poses a challenge for the detection and the real-time monitoring of novel antimicrobial resistance trends using molecular and whole genome sequencing-based methods. The aim of our study was to understand cryptic resistance determinants responsible for the phenotypic carbapenem resistance observed in strains circulating in Italy by using a combined approach involving whole genome sequencing (WGS) and genome-wide association study (GWAS). In this study, we collected 303 *Kp* strains from inside and outside clinical settings between 2018–2022 in the Abruzzo region of Italy. The antimicrobial resistance profile of all isolates was assessed using both phenotypic and bioinformatic methods. We identified 11 strains resistant to carbapenems, which did not carry any known genetic determinants explaining their phenotype. The GWAS results showed that incongruent carbapenem-resistant phenotype was associated specifically with strains with two capsular types, KL13 and KL116 including genes involved in the capsule synthesis, encoding proteins involved in the assembly of the capsule biosynthesis apparatus, capsule-specific sugar synthesis, processing and export, polysaccharide pyruvyl transferase, and lipopolysaccharide biosynthesis protein. These preliminary results confirmed the potential of GWAS in identifying genetic variants present in KL13 and KL116 that could be associated with carbapenem resistance traits in *Kp*. The implementation of advanced methods, such as GWAS with increased antimicrobial resistance surveillance will potentially improve *Kp* infection treatment and patient outcomes.

## 1. Introduction

*Klebsiella pneumoniae* (*Kp*) is one of the most common causes of hospital-acquired infections, and it is recognized by the World Health Organization (WHO) as a “critical” priority pathogen for the development of novel antimicrobial strategies (Martin et al., 2021). In particular, the rapid emergence of *Kp* isolates resistant to last-line antimicrobials, such as carbapenems, poses substantial challenges for the treatment of both hospital and community-acquired infections (Navon-Venezia et al., 2017). Indeed, the presence of multidrug-resistant (MDR) *Kp* isolates in diverse environmental niches, even outside the clinical setting, poses a challenge to the detection and real-time monitoring of novel antimicrobial resistance (AMR) trends (Wyres and Holt, 2016).

Limited data are available on *Kp* isolates outside the clinical setting, but Rodrigues et al. (2022) highlighted that transmission can occur between clinical settings and MDR strains from foods. Moreover, Chiaverini et al. (2022) reported the presence of one MDR strain from a wild boar in Italy.

In 2013, Italy was, for the first time, classified as endemic for carbapenem-resistant *Enterobacteriaceae* (CRE) based on the high frequency of hospital-admitted patients carrying resistant strains acquired from autochthonous sources. Among CRE strains, the number of *Kp* strains causing invasive disease has been growing at an alarming rate, and in 2017, Italy was the country with the second-highest percentage of invasive *Kp* isolates with resistance to carbapenems (European Centre for Disease Prevention and Control, 2019). This aspect was confirmed in the National Report on Medicines Use in Italy for 2020 (Italian Medicines Agency, 2021). On the other hand, the occurrence of CRE in livestock in the EU remains low (European Centre for Disease Prevention and Control, 2019).

Carbapenems, including ertapenem and meropenem, are widely used because of their exceptionally broad-spectrum activity and their efficacy in the treatment of complicated intra-abdominal and skin and skin structure infections, nosocomial pneumonia, complicated urinary tract infections, meningitis (meropenem only), and febrile neutropenia (Zhanel et al., 2007). Recently, the association of two synergistic carbapenems (ertapenem plus either meropenem, doripenem, or imipenem), alone or combined with other antibiotics, has been proposed in the treatment for nosocomial meningitis associated with abscesses and drainage (De Pascale et al., 2017). Infections due to carbapenem-resistant *Kp* (CR-*Kp*) are a serious challenge for physicians due to the scarce therapeutic options available, long hospital and intensive care unit (ICU) stays, and consequent increased costs of care (De Pascale et al., 2017). These data arouse concern, given the impact of the use of carbapenems on the further spread of AMR.

Resistance to carbapenems is mediated by a primary mechanism represented by the production of β-lactamases. More recently, carbapenemases, mainly *Kp* carbapenemases (KPCs) and metallo-β-lactamases (MBLs), have become more prevalent mechanisms for CR-*Kp*. KPC producers have been found almost everywhere and are mostly imported from endemic areas in Europe, such as Greece and Italy (Nordmann and Poirel, 2014).

In addition, Ma et al. (2021) reported that the presence of extended spectrum β-lactamases (ESBL) (e.g., *bla*_SHV-27_) and AmpC genes (e.g., *bla*_DHA-1_) could provide some protection for the bacteria against one or more types of carbapenems driving high-level resistance to these compounds.

The other mechanisms responsible for resistance to carbapenems involve changes in membrane permeability restricting antibiotic entry as a result of loss or mutation of specific outer membrane porins (*omp*), in particular, *ompK35/36* (Vergalli et al., 2020).

Although the main carbapenem resistance determinants have been well studied in the clinical setting, potential cryptic mechanisms could still be unknown outside the nosocomial environment and community-acquired infections.

To develop tools for AMR prediction, whole genome sequencing (WGS) of bacterial strains is necessary to collect data about the dynamics of various *Kp* resistance determinants in different hosts and environments. To address this gap, genome-wide association study (GWAS) can be performed to reveal novel associations between phenotype and genotype, as shown by different authors (Farhat et al., 2019; Bokma et al., 2021; Weber et al., 2021; Pei et al., 2022).

The aim of the present study is to understand cryptic resistance determinants responsible for the inconsistencies between phenotypic and genotypic profiles of resistance to carbapenems observed in *Kp* strains circulating in Italy, even outside the clinical setting, using WGS and GWAS methods.

## 2. Materials and methods

### 2.1. Bacterial isolates

In this study, we collected 303 *Kp* strains detected in foods (*n* = 106); feed (*n* = 8) environmental samples, including wastewater and seawater (*n* = 94); animals (*n* = 45); and clinical cases (*n* = 50) between 2018–2022 in the Abruzzo region in central Italy (Supplementary Table S1). All isolates were identified as *Kp* by matrix-assisted laser desorption ionization-time of flight (MALDI-TOF) mass spectrometry using MALDI Biotyper (Bruker Daltonik, Germany) or Vitek MS (Biomérieux, Marcy l’Étoile, France) and confirmed by WGS.

*Kp* isolates from wild animals were collected in the framework of regional plans of epidemiological surveillance and monitoring of diseases in wild fauna, in the frame of the national plan of surveillance of West Nile disease and Usutu for bird species (Chiaverini et al., 2022). In food, food-producing environments, and feed, *Kp* isolates were detected during monitoring plans for foodborne pathogens.

Finally, clinical isolates were collected in a local healthcare unit of the Abruzzo region. The strains were isolated from different specimens, including respiratory secretions, blood, urine, feces, wound swabs, and rectal and vaginal swabs, which were performed during routine diagnostic activities.

### 2.2. Ertapenem and meropenem susceptibility testing

Antimicrobial susceptibility versus ertapenem and meropenem was tested using the broth microdilution method and the Kirby-Bauer disk diffusion test following the EUCAST guidelines [European Society of Clinical Microbiology and Infectious Diseases (EUCAST), n.d.]. AST by broth microdilution was performed using the Vitek 2 (Biomérieux, Marcy l’Étoile, France) or DxM MicroScan WalkAway (Beckman Coulter, Inc., United States) systems or with GN3F plates (Thermo Fisher Scientific, Lenexa, KS), using the Sensititre OptiRead Automated Fluorometric Plate Reading System (Thermo Fisher, Lenexa, KS) as a reading system. A disk diffusion test was carried out using ertapenem (10 μg) and meropenem (10 μg) disks (Oxoid Ltd., Basingstoke, United Kingdom). *Escherichia coli* ATCC25922 was used as a control.

A full description of strains and methods used and a list of Minimum Inhibitory Concentrations (MIC) are given in Supplementary Table S2.

### 2.3. Whole genome sequencing

DNA extraction of *Kp* isolates was carried out according to Portmann et al. (2018). Bacterial cultures were grown overnight on nutrient agar (Microbiol & C., Cagliari, Italy) at 37 ± 1°C. DNA extraction was performed from single colonies using the DNeasy Blood and Tissue Kit (Qiagen, Hilden, Germany) according to the manufacturer’s instructions, and 1 ng of DNA of each sample was used for library preparation using Nextera XT DNA kit (Illumina, San Diego, CA, United States), according to the manufacturer’s protocols. Whole genome sequencing was performed using the NextSeq 500 platform (Illumina, San Diego, CA, USA) with the NextSeq 500/550 mid-output reagent cartridge v2 (300 cycles, standard 150-bp paired-end reads).

For the WGS data analysis, an in-house pipeline was used (Cornacchia et al., 2022). The genome assembly quality check was assessed according to Hennart et al. (2022) (n. contigs < 1,000, total length ranged from 4.5 to 6.5 Mbp, GC% < 59%). The species confirmation and K/O locus determination were performed in Kleborate (Lam et al., 2021) hosted on the Pathogenwatch platform (Argimón et al., 2021); meanwhile, sequence type (ST) was calculated *in silico* according to the multilocus sequence typing (MLST) scheme hosted in the Pasteur sdb platform (Diancourt et al., 2005; Brisse et al., 2009).

Chromosomal and acquired ESBLs, β-lactams and carbapenems resistance genes, and outer membrane porin alterations were detected querying the ResFinder 4.1 database (v. 21st February 2023) (Florensa et al., 2022) with a default threshold of 90% for %ID and 60% minimum length.

The genome assemblies were deposited in DDBJ/ENA/NCBI SRA repository under the BioProject PRJNA1001640 and PRJNA774508.

### 2.4. Genome-wide association studies

The genome annotation of all 303 *Kp* genomes was carried out using Prokka (Seemann, 2014) to produce GFF-files, using a reference genome (CP003200.1), the same GFF-files were used to extract the pangenome with Panaroo (Tonkin-Hill et al., 2020). The GWAS (Lees et al., 2018) was performed to identify genes significantly associated with inconsistent carbapenem phenotypic resistance. In order to control for the confounding population stratification, a pairwise comparison based on a linear mixed model was performed using an ML tree from the CFSAN pipeline (Davis et al., 2015) using CP003200.1 as a reference for the variant calling. The visualization of the ML tree was performed using Interactive Tree of Life (iTOL).^1^

Information regarding the obtained significant genes was retrieved using BLAST.^2^

## 3. Results

### 3.1. Ertapenem and meropenem susceptibility testing

Out of 303 *Kp* strains collected in this study, 263 *Kp* isolates were found to be susceptible to meropenem and ertapenem; meanwhile, a total of 40 *Kp* were phenotypically resistant to at least one type of carbapenem, as reported in Supplementary Table S3. A total of 23 were resistant to ertapenem and meropenem, 14 only to ertapenem, and 3 only to meropenem. As expected, the majority of resistant *Kp* strains (*n* = 29) were isolated in the clinical setting from various specimens (blood, feces, respiratory secretions, urine). The remaining 11 carbapenem-resistant strains were collected from poultry meat products (chicken legs *n* = 2 and hamburgers *n* = 2) and wild animals: one European badger (*Meles meles*), two fallow deers (*Dama dama*), one magpie (*Pica pica*), one red deer (*Cervus elaphus*), and two wild boars (*Sus scrofa*).

### 3.2. Genomic characterization of *Kp* population

Taxonomic analysis confirmed all the strains as *Kp*. According to the MLST scheme hosted in Pasteur, 150 STs were identified, including 20 STs as new, each corresponding to a single genome (Supplementary Table S1). The most abundant STs were ST35 (*n* = 16), ST45 (*n* = 15), ST37 (*n* = 11), and ST307 (*n* = 10). The most common STs within clinical cases were represented by high-risk clones ST307 (*n* = 10), ST512 (*n* = 9), and ST101 (*n* = 5). Querying the Pathogenwatch platform, 16 different O loci were the most representative as O1v1 (*n* = 50), O2v1 (*n* = 42), and O3b (*n* = 45), and 79 different capsular types (KL) were identified as reported in Supplementary Table S1.

Querying the ResFinder 4.1 database, carbapenemases resistance genes (*bla*_KPC-3_ and *bla*_KPC-9_) were identified in 30 clinical isolates. ESBLs resistance genes *bla*_CTX-M-15_ were identified in 19 isolates from humans (*n* = 9), chicken meat products (*n* = 5), environment (*n* = 4, one seawater and 3 wastewater), and one wild boar; *bla*_OXA_ variants (*bla*_OXA-1_ and *bla*_OXA-9_) were harbored by 37 strains from humans (*n* = 24), wild animals (*n* = 8) and environment (*n* = 5). Other ESBLs *bla* variants, *bla*_SHV-27_ and *bla*_SHV-41_, were highlighted in 27 strains, all collected from outside the clinical setting, and *bla*_DHA-1_, a plasmid-borne inducible AmpC gene, in three strains from chicken meat products. Instead, other β-lactams resistance genes were detected in 265 of 303 isolates.

**Figure 1:**
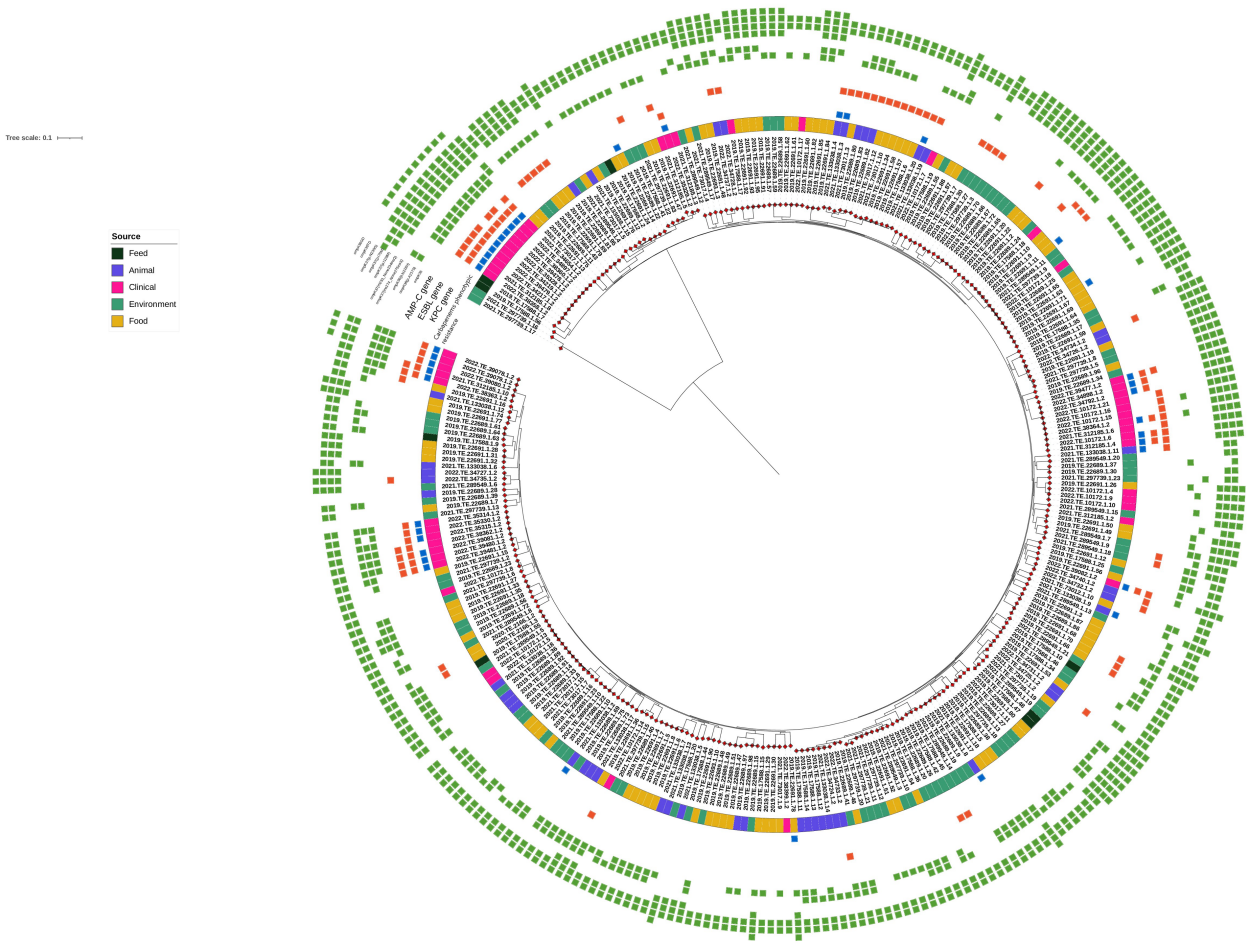
Maximum Likelihood (ML) midpoint rooted tree obtained from CFSAN pipeline. The first layer represents the source of isolation as shown in the legend. Carbapenems phenotypic resistance (blue square), *bla*_KPC_, ESBL and AmpC genes (red square), and *ompK* mutation variants (green square) are shown in the heatmap. Gene profiles and gene presence/absence were visualized using Interactive Tree of Life (iTOL) (https://itol.embl.de/).

Checking for outer membrane porin alterations, several *ompK36* and *ompK37* point mutations were identified in the entire dataset; moreover, *ompK35* truncations were detected in 26 strains, of which 23 were of clinical origin, one was from chicken meat, and two were from wastewater. Finally, the presence of *ompK36* with a glycine-aspartate (GD) or threonine-aspartate (TD) L3 insertion was found in 13 *Kp* genomes from carbapenem-resistant strains isolated from human samples. All results are summarized in Figure 1.

We detected several plasmid replicons, including Inc-type and Col-type replicons that were present in 237 genomes of the dataset.

Among the 40 carbapenems phenotypically resistant strains, 29 clinical strains harbored *bla*_KPC-3_ and *bla*_KPC-9_ genes, and 11 *Kp* isolates detected outside of the clinical setting (7 from wild animals and 4 from chicken meat products) did not carry any known genetic determinants that could account for their resistance to meropenem and ertapenem but harbored several β-lactam resistance genes and some ESBL genes. Indeed, among them, one strain from a chicken product harbored *bla*_DHA-1_ and four strains from wildlife carried the ESBL-variant *bla*_SHV-27_. Point mutations were detected in all the isolates, but only five strains harbored plasmid replicons (Figure 1). All detailed results are reported in Supplementary Table S1.

### 3.3. Identification of significant genes

GWAS analysis was conducted to understand which genes might be involved in the inconsistent carbapenem-resistant phenotype found in the 11 *Kp* genomes.

From the predicted pangenome, we identified a core of 4,148 Coding Sequences (CDSs) present in each of the sequenced isolates, 115 soft-core genes (i.e., in more than 95% of the genomes), 1,227 shell genes present from 15 to 95% of the genomes, and 12,235 different genes in less than 15% of the genomes.

**Figure 2:**
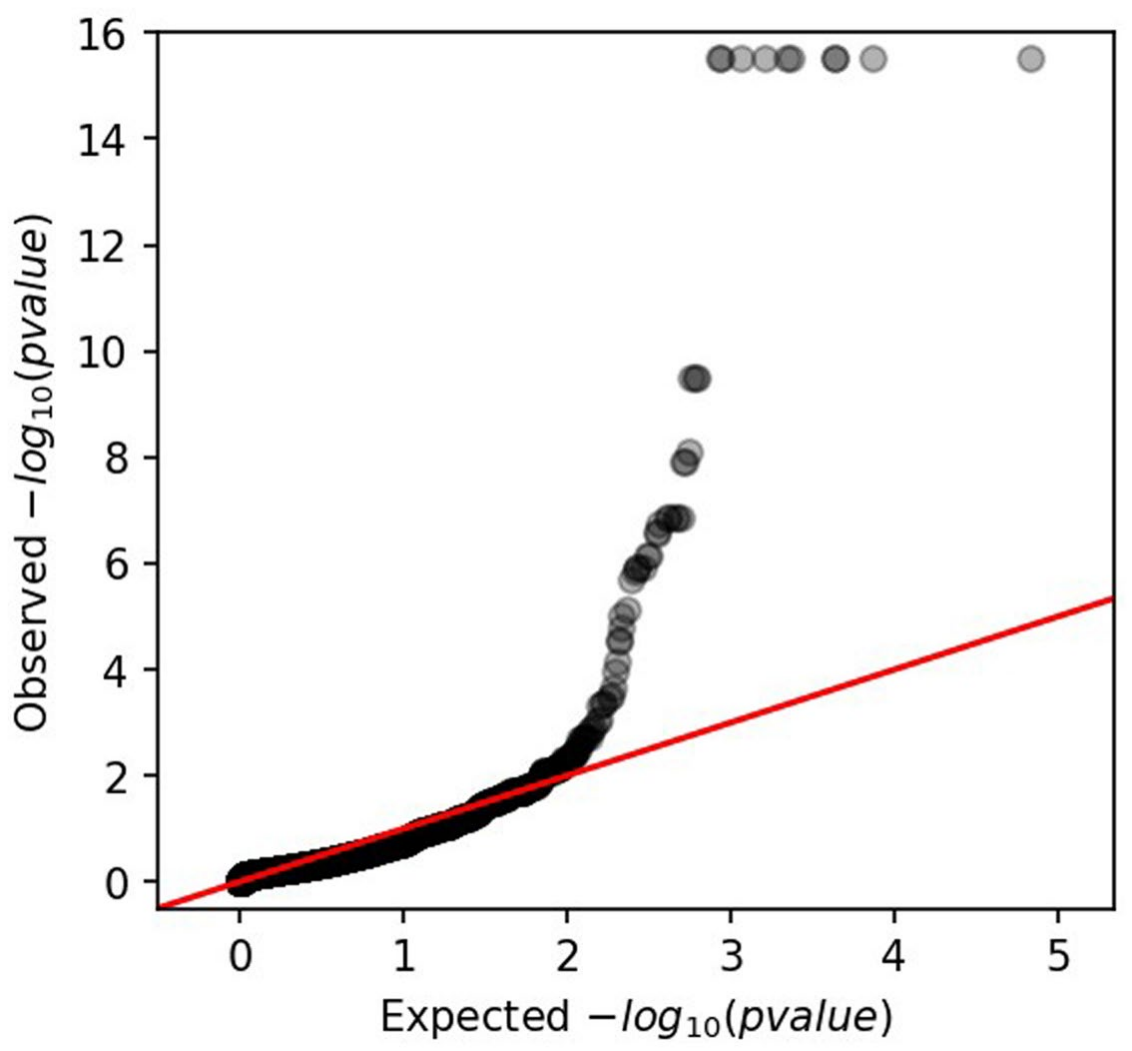
QQ-PLOT obtained from GWAS analysis representing the lrt value of *ps* (the value of *p* from the linear mixed model association).

Applying an lrt-*p* value equal to 7.02E-06, 31 significant genes were identified among the annotation genes. According to the QQ PLOT (Figure 2), the GWAS revealed 12 genes with high lrt-*p* values (from E-10 to E-16) showing to be highly significant and involved in capsule polysaccharide assembly and export. All those genes and their inference functions are reported in Supplementary Table S4. In particular, the genes encode polysaccharide pyruvyl transferase, lipopolysaccharide biosynthesis protein, general stress protein, glycosyltransferases, O- antigen and lipid-linked capsular repeat unit polymerase, O- flippase, tyrosine-protein kinase, and two hypothetical proteins.

Checking the presence-absence of the 12 significant genes in all genomes, two patterns were detected. The first pattern was the presence of polysaccharide pyruvyl transferase and lipopolysaccharide biosynthesis coding genes and was detected in six genomes belonging to KL116.

The second pattern, however, was characterized by a genetic cassette consisting of nine genes involved in capsule synthesis and one phage tail coding gene and was detected in four genomes belonging to KL13.

The two patterns were present in both resistant and susceptible strains. Indeed, the first pattern was found in three CR-*Kp* (detected in wild animals) and in three susceptible strains isolated from raw milk and chicken meat products. The second one, instead, was identified in three CR-*Kp* (one chicken meat product and two wild animals) and one susceptible strain isolated from a roe deer (*Capreolus capreolus*). Furthermore, the genes of both patterns were found in 6 of the 11 inconsistent carbapenem-resistant strains; meanwhile, in five genomes, no genes belonging to pattern 1 or 2 were identified.

## 4. Discussion

The results of our work reveal high genomics diversity in the *Kp* population analyzed, in particular, outside the clinical setting. The presence of high-risk clones ST35, ST37, ST45, and ST307, mostly associated with humans, was observed also outside the clinical setting, mainly in food samples, but also in animals and in the environment. Our results also confirm the predominance of CR-*Kp* strains in samples collected from hospitalized patients (29, 58%), conferred by the presence of *bla*_KPC_ genes. Indeed, within the clinical setting, the presence of a more homogeneous population, mostly represented by high-risk clones ST307, ST512, and ST101, is consistent with the population structure of CR-*Kp* strains observed in Italy (Rimoldi et al., 2017; Fasciana et al., 2019; Di Pilato et al., 2021; Di Mento et al., 2022).

The presence of carbapenem-resistant *Kp* strains observed in wild animals and chicken meat products instead revealed a non-specific and perhaps accidental mechanism, unrelated to the expression of known carbapenemases.

The high consumption of antibiotics in human and veterinary sectors puts Italy, together with Greece, among the European countries with the highest prevalence of antibiotic-resistant bacteria; in particular, the burden of CR-*Kp* increased the most in terms of number of infections and deaths (Cassini et al., 2019). In accordance with European Medicine Agency (2019), the use of carbapenems is not authorized in veterinary medicine, especially in food-producing animals, and their use is allowed only in exceptional cases in pet animals. Therefore, the observed resistance to those antimicrobials in strains detected in animals or food could be a result of the acquisition of resistance factors directed against other β-lactams or environmental contaminants, such as pesticides and disinfectants (Squadrone, 2020; Giacometti et al., 2021).

The role of the environment in the promotion and dissemination of carbapenemase genes was previously described by Mills and Lee (2019), who showed that transmission was predominantly linked to anthropomorphic activities (in the hospital and municipal wastewater and agricultural environments), and to pet animals, wildlife, livestock, and retail food products. Furthermore, the most prevalent carbapenemase-producing gene in animals was *bla*_OXA_, which may indicate that the strains carrying this gene have high potential for transmission from animals to humans, even if the pathway of transmission is not well defined (Guerra et al., 2014). Indeed, the *bla*_OXA_ genes were harbored by *Kp* strains isolated also from the human and environmental samples of our dataset, in particular from wastewater and seawater samples, suggesting that the dynamics of transmission are complex and the contribution of human activities to the spread of antimicrobial resistant bacteria and/or antimicrobial resistance genes is difficult to understand. The available evidence suggests that *Kp* is a prime target for the sentinel surveillance of AMR thanks to its ability to move between and proliferate in multiple ecological niches, also providing more opportunities for genetic exchange with a wide range of bacterial species (Wyres and Holt, 2018).

Carbapenem resistance, however, does not only depend on the presence of carbapenemases genes but may also be conferred by the combination of alternative mechanisms, such as the action of ESBL or AmpC enzymes, together with decreased membrane permeability and/or the over-expression of efflux pumps (Goessens et al., 2013; Guerra et al., 2014; van Boxtel et al., 2016; Tseng et al., 2023).

Here, in 5 of the 11 strains of animal origin showing resistance to at least one type of carbapenem but not carrying carbapenemase genes, genes coding for extended spectrum or AmpC β-lactamases were detected. In particular, in four genomes, we identified the *bla*_SHV-27_ gene and in one genome we detected *bla*_DHA-1_. The *bla*_SHV-27_ was also detected in 20 strains susceptible to carbapenems, suggesting that another mechanism, possibly in conjunction with the ESBL production, was involved in the expression of carbapenem-resistant phenotypes (Carvalho et al., 2021). Interestingly, only the strain, that harbored *bla*_DHA-1_ showed resistance to both meropenem and ertapenem, while the other ten strains were resistant to only one of these antimicrobials. *Kp* carrying *bla*_DHA-1_ has been previously associated with ertapenem resistance in the absence of the genes coding for carbapenemases (Realegeno et al., 2021).

The *ompK* mutations and the presence of flow pumps could provide important information about the possible multifactorial and non-specific mechanisms of resistance to carbapenems found in food-producing animals and wildlife. Indeed, multiple mutations in *ompK36* and *ompK37* previously associated with decreased resistance to carbapenems were detected, and in some strains, deletions in *ompK35* genes were also detected. Similar patterns of mutations were observed in strains resistant and susceptible to carbapenems and, therefore, could not by themselves be responsible for the observed AMR phenotypes.

A combination of specific mutations in *ompK35* and *ompK36* with ESBL and AmpC expression could be responsible for carbapenem resistance in only five of our *Kp* strains with unexplained resistance phenotype, as demonstrated by Hamzaoui et al. (2018). However, the remaining six strains did not carry this type of β-lactamases. Therefore, we applied GWAS analysis to understand whether the presence or absence of other genes that had not been previously associated with carbapenemase resistance could explain the inconsistent carbapenemase phenotype in some *Kp* strains.

Devanga Ragupathi et al. (2020) and Pei et al. (2022) used GWAS to identify genetic modifications linked to carbapenemase resistance in *Kp* strains isolated from clinical samples, concerning our work, strains isolated outside the clinical context, could be subjected to different environmental selection pressures, leading to different antimicrobial resistance mechanisms.

Furthermore, unlike Pei et al. (2022) who analyzed the mechanisms related to resistance to carbapenems (imipenem and meropenem), our work has focused on understanding the inconsistencies found between the results obtained from the phenotype analysis compared to those obtained from the whole genome analysis.

GWAS analysis of our dataset showed that the incongruent carbapenem-resistant phenotype was associated specifically with strains with two capsular types, KL13 and KL116. In KL13, the significant GWAS hits included nine genes from the K-locus linked to capsule synthesis, encoding proteins involved in the assembly of the capsule biosynthesis apparatus, capsule-specific sugar synthesis, processing, and export (Wyres et al., 2016). To our knowledge, the presence of any type of *Kp* capsule has not been directly associated with changes in antibiotic resistance. The capsule, however, has been shown to provide protection against antimicrobial immune peptides, complement-mediated killing, or from phage infection (Merino et al., 1992; Campos et al., 2004; Wyres et al., 2016). Recently, Ma et al. (2021) examined the *in vitro* evolution of carbapenem resistance in *Kp* in response to antibiotic treatment and showed that mutation in the K-locus gene *wzc*, involved in exopolysaccharide synthesis, leads to a two-fold increase in ertapenem resistance. This could suggest that specific variants of K-locus genes could determine the increase or decrease in the MIC for carbapenems. Ernst et al. (2020) also highlighted that *wzc* mutations could lead to hypercapsule production in carbapenem-resistant strains; therefore, this aspect could be investigated more.

Moreover, polysaccharides, glycans, and glycoconjugates are synthesized by dedicated glycosyltransferases (GTs), a family of enzymes able to transfer a specific carbohydrate residue from a nucleotide- or lipid-pyrophosphate-activated donor to a certain carbohydrate, lipid, or protein acceptor substrate. GTs enzymes, included in our significant GWAS hits, are all involved in the production of bacterial structures, which are important in the pathogenicity process (Fulton et al., 2016). Interestingly, glycosyltransferase plays a role in antimicrobial resistance, causing structural alterations impairing target binding (Wright, 2005).

In KL116 genomes, GWAS analysis detected two significant hits that included genes coding for a polysaccharide pyruvyl transferase and a lipopolysaccharide biosynthesis protein. A study has shown that modifications in outer membrane lipopolysaccharide structure in Gram-negative bacteria, including alterations in LPS anchor-lipid A, can induce tolerance to carbapenems in the absence of specific β-lactamases targets (Murtha et al., 2022).

It is important to highlight that for both KL types observed, the GWAS significant hits corresponded to genes that were founded in both carbapenem-resistant and susceptible strains. This could suggest that the inconsistent phenotype found was not directly related to the presence of the detected candidate genes but possibly to specific gene variants present in KL13 and KL116 carbapenem-resistant strains.

A strength of our study is its investigation of carbapenem-resistant isolates from ecological niches outside of clinical settings, such as food, environment, and animal sources that are not well known. At the same time, the study has some limitations as it was carried out on a limited number of genomes and represents a collection of isolates from a restricted geographic area. In the future, it will, therefore, be necessary to expand the dataset used in order to validate these preliminary results and extend the GWAS analysis to include not only the detection of the presence/absence of genes but to also identify specific mutations that could be associated with modified susceptibility to carbapenems. However, precautions must be taken to prevent the detection of spurious associations using the GWAS approach, but the implementation of advanced methods together with increased AMR surveillance could potentially improve *Kp* infection treatment and patient outcomes.

## 5. Conclusion

In this study, we described a *Kp* population characterized by high genomic diversity and the presence of CR-*Kp* strains that can circulate between different settings. We described the presence of strains with phenotypic resistance to carbapenems in food and wild animals, with the possibility of being transmitted to humans. The carbapenem-resistant phenotype of those strains with no evident genomic resistance determinants, such as carbapenemase genes, can be linked to a synergic combination of other mechanisms, so there is an urgent need to understand cryptic and complex resistance mechanisms associated with AMR and, subsequently, to improve antimicrobial therapy. Our preliminary results confirm the potential of GWAS to identify genetic variants that could be associated with antibiotic resistance traits in *Kp*.

The study of the association of specific capsular types with increased resistance to specific antimicrobials could shed more light on the evolution of carbapenem resistance in *Kp*. Moreover, in the context of One Health, it is necessary to further analyze the resistant isolates from food, environment, and animal sources, as these strains may carry novel resistance determinants leading to carbapenem tolerance that can be passed on to humans.

## Data availability statement

The datasets presented in this study can be found in online repositories. The names of the repository/repositories and accession number(s) can be found in the article/Supplementary material.

## Author contributions

ACh, ACo, and AJ conceptualized the study, carried out the bioinformatics analysis, and analyzed the data, organized the draft, and wrote the manuscript. ACo, GC, MS, and SR carried out sample collection, identification, and isolation experiments. ACo, GC, MA, and CC carried out the WGS experiment. ACh supervised the entire work. FP acquired funding and revised the manuscript. All authors contributed to the article and approved the submitted version.

## Funding

This work was supported by the Fondazione Tercas in the framework of the ProDiaco project (Convenzione Rif. Int. 2021.0077).

## Conflict of interest

The authors declare that the research was conducted in the absence of any commercial or financial relationships that could be construed as a potential conflict of interest.

## Publisher’s note

All claims expressed in this article are solely those of the authors and do not necessarily represent those of their affiliated organizations, or those of the publisher, the editors and the reviewers. Any product that may be evaluated in this article, or claim that may be made by its manufacturer, is not guaranteed or endorsed by the publisher.

## Footnotes

^1^ https://itol.embl.de/

^2^ https://blast.ncbi.nlm.nih.gov/Blast.cgi
